# Drawing on Israel’s Experience Organizing Volunteers to Operationalize Drive-Through Coronavirus Testing Centers

**DOI:** 10.1017/dmp.2020.104

**Published:** 2020-04-16

**Authors:** Edward Kim

**Affiliations:** Medical School for International Health at Ben Gurion University of the Negev, Beer Sheva, Israel

**Keywords:** coronavirus, drive-through, COVID-19, screening, Israel

## Abstract

To increase the country’s capacity to test and track suspected coronavirus disease 2019 (COVID-19) cases, Israel launched drive-through testing centers in key cities, including Tel Aviv, Jerusalem, Be’er Sheva, and Haifa. This article examines the challenges that the national emergency medical services and volunteers faced in the process of implementing drive-through testing centers to offer lessons learned and direction to health-care professionals in other countries.

Israel’s Ministry of Health confirmed their first case of coronavirus disease 2019 (COVID-19) on February 21, 2020, following the repatriation of 1 of the 11 passengers aboard the Diamond Princess cruise ship.^1,2^ One month later, the first related death was made public along with details of over 700 confirmed cases.^3^ By the end of March, the number of cases had increased to over 4500.^4^ To respond to the increasing prevalence and spread of coronavirus throughout the country, there was an urgent need to increase testing of suspected individuals and trace community spread.^5^

Working alongside the Ministry of Health, Magen David Adom (MDA)—Israel’s emergency medical, disaster, ambulance, and blood bank service—operationalized drive-through coronavirus testing centers, mirroring the models used by China and South Korea.^6^ Drive-through testing centers, referred to in Israel as “drive and test” facilities, allow for increased testing throughput while maintaining social distancing guidelines.^7^ The process starts after people with symptoms of COVID-19 are instructed to contact MDA’s dispatch center, where they are screened by a physician, given an appointment if necessary, and then instructed to fill out electronic medical forms. Following this registration process, patients receive a QR code to their mobile device to be scanned at the facility on the day of testing. Those who lack access to a car or are unable to travel receive a home visit, where they are tested by MDA staff.

After the first “drive and test” facility was piloted on March 23 in Tel Aviv to test capacity and refine operations, additional facilities were opened in Jerusalem, Be’er Sheva, and Haifa. The following week, mobile testing centers were set up in Tamra, Modi’in-Maccabim-Re’ut, Wadi Ara, and Rahat, the largest Bedouin community in Israel.^8^ Facilities were set up in large open spaces, such as the parking lots of convention centers, stadiums, parks, and markets. The testing centers are staffed with medical teams, police officers, security personnel, and volunteer students, and can process 6 lanes of cars in parallel, taking on average 3-5 min per car.

The medical teams consist primarily of paramedics who oversee operations, police officers and security personnel who direct traffic, and volunteers who don personal protective equipment (PPE) while testing individuals. Volunteer efforts were coordinated by the Federation of Israel Medical Students (FIMS), which enlisted volunteers from a combined pool of approximately 700 medical students in their clinical studies.^9^

## IDENTIFYING PROBLEMS

While the initial setup and deployment of the “drive and test” facilities were executed swiftly and strategically, there were outstanding issues associated with preparation, implementation, and ongoing operations that needed to be addressed.

Anticipating an increased demand on the country’s emergency call center, which typically receives 5500 calls per day, other independent call centers were repurposed and additional centers were set up in schools to expand capacity and respond to 25,000 calls per day. Concurrently, over 200 volunteers signed up and underwent training to staff the multiple “drive and test” facilities and scale operations. However, to comply with health policies, students were trained in small groups of less than 10, which created time lags and constraints on the quality of the training they received. In more detail, PPE protocol reviews were limited to demonstrations only, leaving many students to don PPE for their first time on-site. A further layer of confusion arose from the fact that “drive and test” facilities and home visits used different PPE equipment and protocols.

With respect to communication, several days after the launch of the “drive and test” facilities, FIMS changed the platform volunteers use to sign up for and manage their shifts. Ultimately, this left many volunteers unable to log into the new system and view available shifts. This problem was compounded by the fact that the team relied on a single WhatsApp group, of over 250 participants, to disseminate all information related to volunteering. Although it was helpful to access a centralized resource, unless volunteers were checking their messages constantly, information was quickly lost due to the steady stream of new messages.

Not only did volunteers face challenges related to training and staffing during the preparation and early implementation phases, they also confronted operational issues during the first 2 wk of launch. For example, volunteers were not given a script to reference before testing individuals. In the first week, a driver stopped their car during testing using only the brake pedal with their car still in gear. Startled during the swabbing process, the driver jerked pressing the accelerator instead and caused an accident. After the incident, volunteers were given a script that, among other things, instructed drivers to put their vehicle in park and engage their parking brake.

More broadly, the rush to set up “drive and test” facilities and coordinate volunteers has had unintended downstream consequences. Laboratories have received viral cultures that are unlabeled or contain inaccurate identification stickers; bags and cultures with identification stickers that do not match; as well as missing or illegible patient information on hand-written forms. Coolers have also been left open for extended periods of time between sample collections. If not quickly addressed, these quality control issues will result in wasted resources, repeat testing, and inaccurate results, putting patients and others at risk if left unresolved.

## ADDRESSING PROBLEMS

Recognizing and responding to shortcomings is critical to successful operations in times of crisis. Accordingly, an anonymous survey was disseminated to collect complaints and recommendations from volunteers to gain insights on outstanding issues.

Fortunately, MDA addressed many of the operational issues quickly and decisively, in some cases overnight. For problems related to limited training, MDA shared updated internal standards and protocols in-person and pushed the same updates to the “MDA teams” app to improve the onboarding process for new volunteers. Subsequently, new volunteers were paired with experienced ones for at least 1 full shift. This was a feasible solution, considering each station already required 2 volunteers: 1 to prepare and log the sample and the other to take the sample from the patient. The pairing strategy helped with oversight between the volunteers without fostering inefficiencies.

It is worth noting that the continued tightening of government-mandated travel restrictions and stricter enforcement of such policies did not reduce MDA and FIMS’ ability to organize volunteers. Both organizations were quick to offer guidance on how to travel to testing sites. While there was no mechanism to provide volunteers with official certificates to expedite travel exceptions, a separate hotline was set up for those who faced any difficulty with law enforcement and to dismiss any accrued fines.

## DISCUSSION

In times of crisis, communication and organizational challenges occur more frequently. Even with ample experience in emergency response and pilot testing, it is not possible to anticipate and prevent every hurdle. An appropriate balance between speed and thoroughness was achieved by MDA’s handling of the “drive and test” facilities.

To quickly operationalize “drive and test” facilities while allowing for process improvements, MDA used the Plan-Do-Check-Act methodology. This enabled the steering team to quickly identify and address areas of improvement while maintaining a balance between iterative refinement and consistency.^10^ Testing center processes evolved over the first 2 wk of implementation and resulted in a largely successful operation. By the end of March, “drive and test” centers had collected over 2000 tests daily, which accounted for 18,000 of the 53,000 tests completed nationwide.^11^
